# Influence of Alloy Composition and Fabrication Techniques on the Marginal and Internal Fit of Fixed Dental Prostheses: A Narrative Review

**DOI:** 10.7759/cureus.113072

**Published:** 2026-07-21

**Authors:** Sukirtha Parasuraman, Jaini JL, Ananjana B Krishnan

**Affiliations:** 1 Prosthodontics, Amrita Institute of Medical Sciences and Research Centre, Kochi, IND

**Keywords:** alloy composition, dental casting alloys, dmls, fixed dental prosthesis, internal fit, lost wax casting, marginal fit, prosthodontics

## Abstract

For individuals who are partially edentulous, fixed dental prostheses (FDPs), such as single crowns and fixed partial dentures, are crucial for restoring function, aesthetics, and structural continuity. Accurate marginal and internal fit are crucial for the long-term success of these restorations because inconsistencies can lead to cement disintegration, plaque buildup, microleakage, secondary caries, periodontal breakdown, and pulpal irritation. These issues can eventually lead to restorative failure, which can include loss of retention or debonding of the restoration, fracture or chipping of the restoration or supporting tooth structure, recurrent infection necessitating restoration replacement, and, in extreme circumstances, the need for more involved procedures like endodontic therapy or tooth extraction. From a therapeutically relevant prosthodontic standpoint, this narrative review sought to assess how alloy composition and fabrication methods affected the marginal and internal fit of FDPs.

Databases like PubMed, Medical Literature Analysis and Retrieval System Online (MEDLINE), and EBSCOhost were used to search the literature for English-language research published between 1990 and 2025. Included were pertinent comparative studies, randomized and non-randomized trials, observational studies, validation studies, in vitro investigations, and review articles assessing titanium, nickel-chromium (Ni-Cr), and cobalt-chromium (Co-Cr) alloys made using direct metal laser sintering (DMLS) and conventional casting methods.

According to the examined data, titanium alloys often exhibit less predictable marginal adaptation than Co-Cr and Ni-Cr alloys. Due to numerous technique-sensitive laboratory procedures, conventional lost-wax casting techniques demonstrated higher variability but produced clinically acceptable results. On the other hand, while internal fit varied based on workflow factors and post-processing procedures, available literature suggests that, especially DMLS and additive manufacturing techniques, may improve reproducibility and marginal accuracy in numerous tests.

Clinically, better internal and marginal adaptation may eliminate chairside adjustments, increase the durability of restorations, and lessen biological problems. Overall, rather than being the product of a single material or fabrication method, restoration fit should be viewed as the consequence of an integrated interplay between alloy composition, fabrication workflow, and laboratory precision.

## Introduction and background

In restorative dentistry, fixed dental prostheses (FDPs) are essential because they provide a long-lasting and stable replacement for lost teeth, limiting the migration of neighboring teeth, maintaining the structural integrity of the dental arch, restoring masticatory function, and greatly improving the patient's quality of life [1]. However, meeting mechanical, biological, and cosmetic requirements is crucial for restorations to be clinically successful and long-lasting. Because they directly affect important elements like marginal integrity, structural stability, and the maintenance of periodontal and pulpal health, marginal and internal adaptations are crucial. Marginal fit is defined as the perpendicular discrepancy between the restoration margin and the finish line of the prepared abutment tooth [2]. Dental biofilm buildup, discoloration, hypersensitivity, marginal leakage, periodontal diseases, and bone loss can all result from increased marginal discrepancy, which can cause cement disintegration [3]. McLean and von Fraunhofer historically reported that a cement film thickness of approximately 120 μm could be considered clinically acceptable [4]. As a result, this value is now commonly regarded as a historical upper threshold, rather than an ideal marginal fit. More recent literature has suggested stricter marginal gap values, typically in the range of 20-75 μm, as the ideal or preferred target for improved marginal adaptation. Therefore, in the present study, marginal discrepancies between 20 and 75 μm were considered optimal, while values up to 120 μm were deemed clinically acceptable but not ideal [5]. In a similar vein, interior fit is critical to the overall effectiveness of FDPs. The length of the perpendicular line drawn from the abutment tooth's surface to the restoration surface is its definition [2]. Under ideal laboratory conditions, the cement film thickness required for complete seating and adequate retention is approximately 20-40 μm [6]. However, depending on the material and prosthesis site, clinically acceptable internal gaps are usually advised to be between 50 and 100 μm [7]. Inappropriate internal fit can result in insufficient retention, impaired resistance, and uneven stress distribution, all of which could jeopardize the structural integrity of the prostheses and the supporting tooth or implant. For FDPs to be durable and useful, a precise marginal and internal fit must be achieved [3].

Different dental alloys exhibit distinct physical, thermal, and metallurgical properties that can influence the marginal and internal fit of FDPs. Variations in alloy composition alter melting range, coefficient of thermal expansion, solidification shrinkage, castability, elastic modulus, and oxide layer formation, all of which affect dimensional accuracy during casting or additive manufacturing [8]. Consequently, prostheses fabricated from different alloys may demonstrate differences in marginal adaptation and internal fit even when identical fabrication protocols are followed. Conventionally, cobalt-chromium (Co-Cr) and nickel-chromium (Ni-Cr) alloys have been widely used because of their favorable mechanical properties, corrosion resistance, and dimensional stability. Titanium has gained increasing attention owing to its excellent biocompatibility, corrosion resistance, and high strength-to-weight ratio; however, its high melting temperature and strong affinity for oxygen make processing more technique-sensitive, which may adversely influence prosthesis fit. Therefore, understanding the influence of different alloy systems on restoration adaptation is essential when selecting materials for FDPs [9].

**Figure 1:**
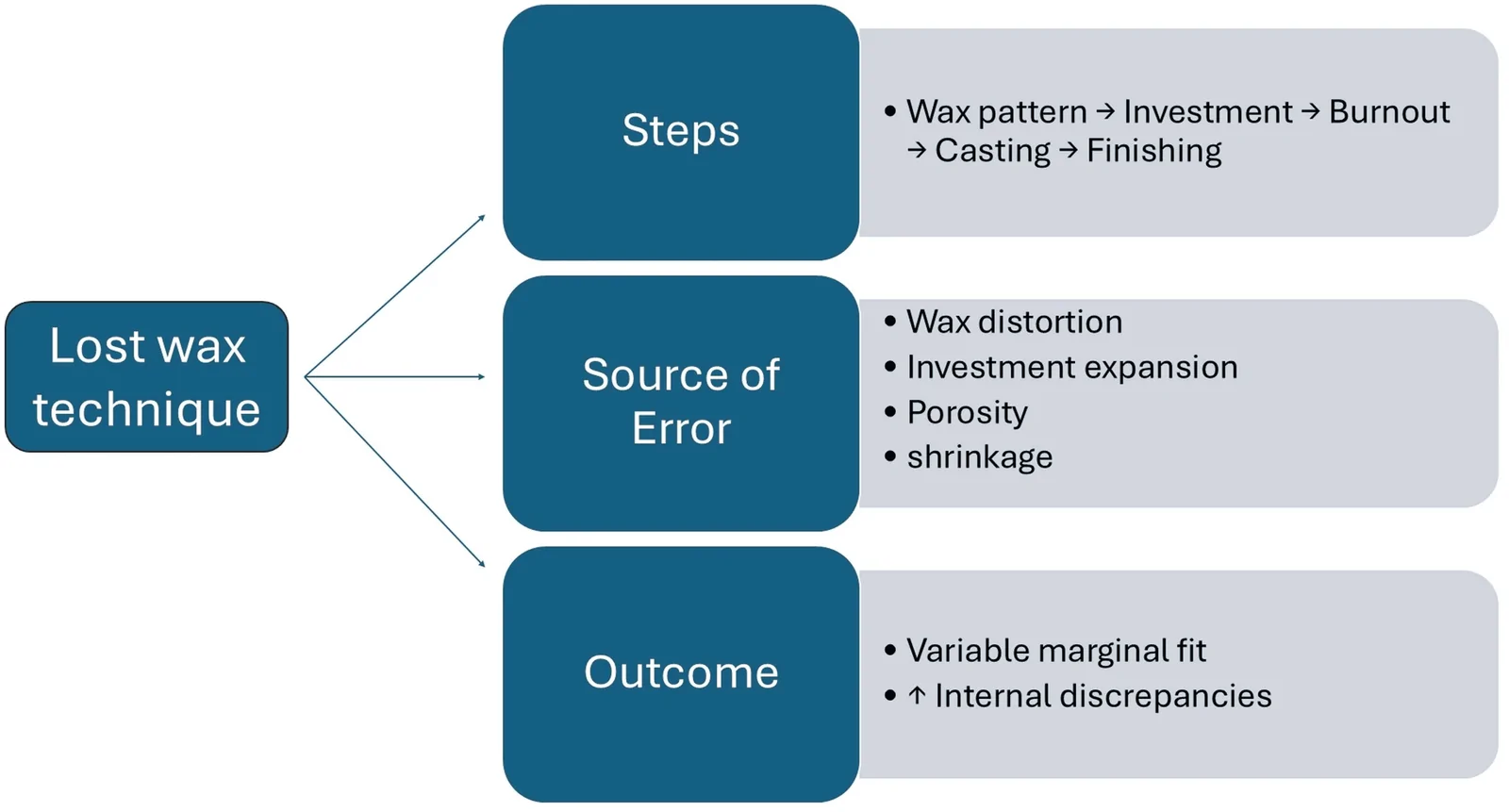
Schematic representation illustrating the fabrication steps of the lost-wax technique, common sources of error that can occur with the lost-wax technique, and their influence on marginal and internal discrepancies. Author-generated schematic illustration of the lost-wax fabrication technique and potential sources of marginal and internal discrepancies. Created by the authors using Microsoft PowerPoint for Microsoft 365 (Version 2505, Microsoft Corporation, Redmond, WA, USA).

**Figure 2:**
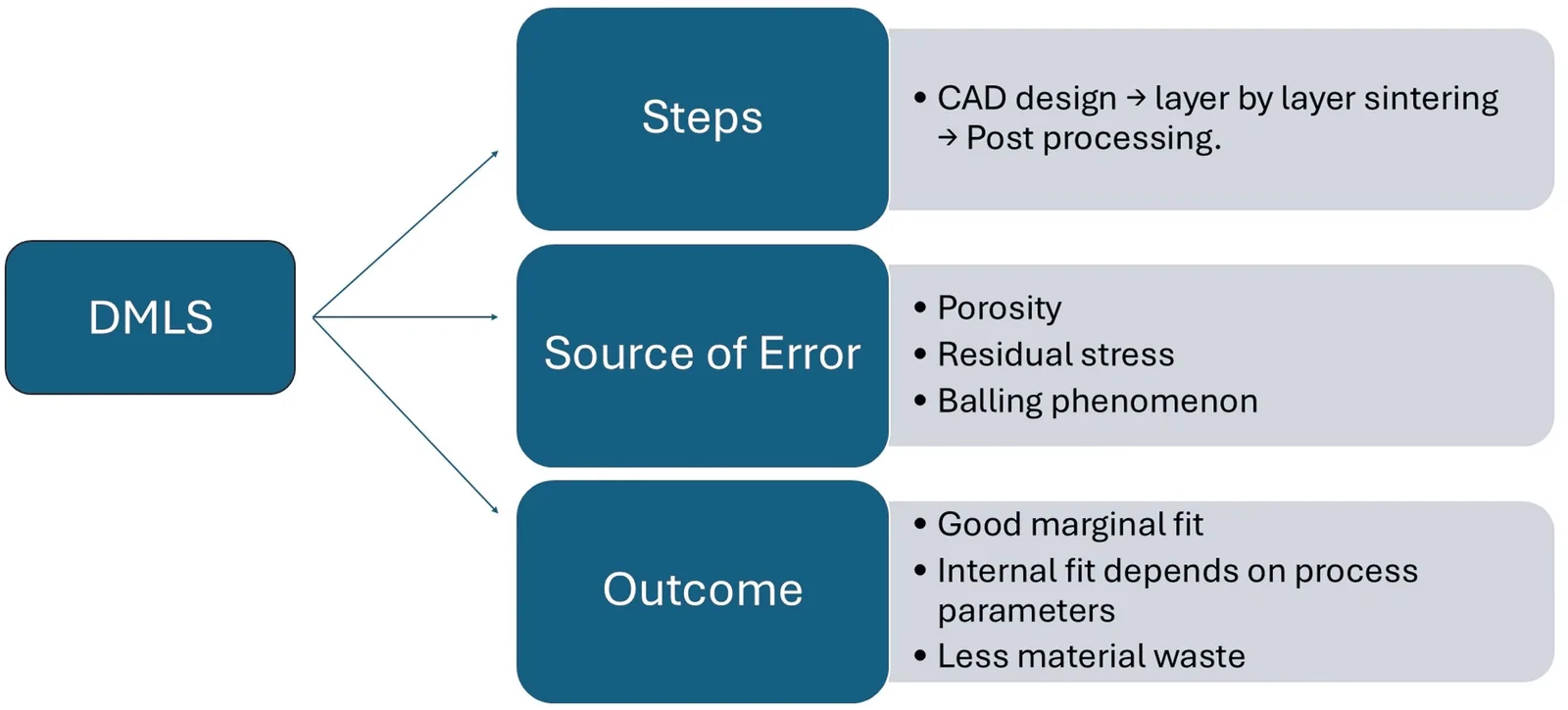
Schematic representation illustrating the fabrication steps of the DMLS technique, common sources of error that can occur with the DMLS technique, and their influence on marginal and internal discrepancies. Author-generated schematic illustration of the DMLS fabrication technique and potential sources of marginal and internal discrepancies. Created by the authors using Microsoft PowerPoint for Microsoft 365 (Version 2505, Microsoft Corporation, Redmond, WA, USA). DMLS: direct metal laser sintering; CAD: computer-aided design

Another important factor influencing the marginal and internal fit of FDPs is the fabrication process. In dental prosthetics, conventional methods like the lost-wax casting method have long been the norm. The conventional lost-wax workflow and the potential sources of dimensional error are illustrated in Figure 1. Dental framework fabrication has undergone tremendous improvement since the introduction of modern manufacturing processes, especially direct metal laser sintering (DMLS). The DMLS fabrication workflow and associated sources of discrepancy are illustrated in Figure 2.

Evidence incorporating these variables in connection to marginal and internal fit is still dispersed, even though much research has assessed individual alloys and production methods. This narrative review's objective was to assess, from a clinically relevant prosthodontic standpoint, the impact of alloy composition and fabrication methods on the marginal and internal fit of FDPs.

## Review

Literature search strategy

A narrative literature review was conducted to evaluate the influence of alloy composition and fabrication techniques on the marginal and internal fit of FDPs. A literature search of English-language publications between 1990 and 2025 was conducted. Databases searched were PubMed, Medical Literature Analysis and Retrieval System Online (MEDLINE), and EBSCOhost, along with additional hand searches. The search strategy included combinations of keywords and MeSH terms such as “fixed dental prosthesis,” “marginal fit,” “internal fit,” “alloy composition,” “conventional casting,” “direct metal laser sintering,” “DMLS,” and “fabrication techniques” coupled with appropriate Boolean operators (AND, OR). Additional relevant articles were identified through manual screening of reference lists. Articles were selected for relevance to the review topic. Comparative studies, observational studies, validation studies, in vitro investigations, and review articles evaluating the effects of alloy composition and fabrication methods on prosthesis adaptation were included. The studies focused on evaluating the effects of alloy composition and fabrication techniques on the marginal and internal fit of FDPs. Digital workflows, such as DMLS, were compared with conventional methods like lost-wax casting. Marginal gaps and internal fits were measured as key indicators of clinical success. The search identified 126 records. After screening titles, abstracts, and full texts for relevance, nine articles were included in the final narrative review. A narrative synthesis of the selected literature was performed to summarize the current evidence of the influence of alloy composition and fabrication techniques on the marginal and internal fit of FDPs. As a narrative review, the objective was to provide an overview of the available evidence rather than to perform a formal systematic review or meta-analysis. Table 1 presents the inclusion and exclusion criteria used for the selection of studies in this narrative review.

**Table 1:** Inclusion and exclusion criteria. DMLS: direct metal laser sintering; CAD: computer-aided design; CAM: computer-aided manufacturing

Included	Excluded
Studies on full metal single crowns up to three-unit bridges fabricated using conventional casting or DMLS techniques; studies evaluating the marginal and internal fit of single crowns up to three-unit bridges fabricated using these techniques	Studies involving bridges with more than three units; studies involving single crowns up to three-unit FPDs fabricated by porcelain fused metal (PFM), zirconia; studies on single crowns up to three-unit bridges fabricated using CAD-CAM; removable prostheses
Randomized controlled trials, non-randomized trials, quasi-experimental studies, validation studies, observational comparative studies, and in vitro studies	Case reports, case series, conference abstracts, letters, and studies with insufficient data
Studies published in English	Studies published in languages other than English
Studies published between 1990 and 2025	Studies published before 1990
Full-text articles available	Studies without full-text access; unpublished thesis

Results

The reviewed literature included studies evaluating Co-Cr, Ni-Cr, and titanium alloys fabricated using conventional casting and DMLS techniques, with marginal and internal fit assessed as indicators of prosthesis adaptation. Among these, five studies evaluated Co-Cr alloys, three studies investigated Ni-Cr alloys, and two studies assessed titanium alloys. Regarding fabrication techniques, six studies evaluated conventional casting techniques, and five studies investigated DMLS/selective laser melting (SLM) workflows. Also discussed were digital fabrication workflows and additive manufacturing concepts in fixed prosthodontics. Most included studies were in vitro comparative investigations evaluating marginal and internal fit as indicators of prosthesis accuracy and clinical acceptability.

Effect of Conventional Casting on Co-Cr Alloys

Bhaskaran et al. (2013), in an in vitro comparative study involving 30 Co-Cr copings, compared conventional wax-pattern casting, resin-pattern casting, and DMLS fabrication techniques. The mean marginal discrepancy was 45.36 µm for wax-pattern casting, 27.22 µm for resin-pattern casting, and 10.52 µm for DMLS. Internal gap values were 39.97 µm, 36.15 µm, and 41.22 µm, respectively. The findings suggested that conventional techniques were more technique-sensitive and associated with greater dimensional inaccuracies [10].

James et al. (2018), in a comparative in vitro study involving 30 Co-Cr copings, evaluated DMLS, computer-aided manufacturing (CAM), traditional casting, and ringless casting techniques. The mean marginal discrepancy was 103.08 ± 7.29 µm for DMLS, 109.46 ± 10.63 µm for CAM, 101.78 ± 7.34 µm for traditional casting, and 98.72 ± 4.57 µm for ringless casting. Internal gap values were not reported. The study concluded that conventional casting demonstrated clinically acceptable adaptation but greater variability because of multiple laboratory procedures [11].

Table 2 summarizes the studies evaluating the effect of conventional casting techniques on the marginal and internal fit of Co-Cr alloy restorations.

**Table 2:** Effect of conventional casting on Co-Cr alloys. DMLS: direct metal laser sintering; CAM: computer-aided manufacturing; Co-Cr: cobalt-chromium; NR: not reported

Author (Year)	Study Design/Sample Size	Alloy Composition/Fabrication Technique	Key Findings	Mean Marginal/Internal Gap (µm)
Bhaskaran et al. (2013) [10]	Comparative in vitro study (n=30)	Co-Cr; conventional wax-pattern casting, resin-pattern casting, DMLS	Conventional fabrication showed a higher marginal discrepancy than DMLS	Marginal: wax 45.36; resin 27.22; DMLS 10.52. Internal: wax 39.97; resin 36.15; DMLS 41.22
James et al. (2018) [11]	Comparative in vitro study (n=30)	Co-Cr; lost-wax casting, DMLS, CAM, ringless casting	Conventional casting demonstrated acceptable adaptation but greater variability	Marginal: DMLS 103.08 ± 7.29; CAM 109.46 ± 10.63; traditional casting 101.78 ± 7.34; ringless casting 98.72 ± 4.57. Internal: NR

Effect of DMLS on Co-Cr Alloys

Bhaskaran et al. (2013), in an in vitro comparative study involving 30 Co-Cr copings, reported improved marginal adaptation in DMLS-fabricated restorations compared with conventional fabrication techniques. The mean marginal discrepancy was 10.52 µm for DMLS, compared with 27.22 µm for resin-pattern casting and 45.36 µm for wax-pattern casting. Internal gap values were 41.22 µm for DMLS, 36.15 µm for resin-pattern casting, and 39.97 µm for wax-pattern casting [10].

Arora et al. (2018), in an in vitro comparative study involving 30 Co-Cr restorations, compared DMLS fabrication, resin-pattern casting, and conventional wax-pattern casting. The mean marginal discrepancy was 78.28 ± 6.98 µm for DMLS, 101.67 ± 4.88 µm for resin-pattern casting, and 107.84 ± 5.63 µm for wax-pattern casting. However, internal discrepancy was greater in the DMLS group (169.38 ± 6.00 µm) than in the resin-pattern (133.87 ± 6.64 µm) and wax-pattern groups (140.84 ± 9.39 µm). These findings indicate that DMLS improved marginal adaptation, although internal fit remained variable [12].

Sharma et al. (2017), in an in vitro study involving 30 Co-Cr copings, compared DMLS fabrication with conventional lost-wax casting. The mean marginal discrepancy was 27.9 ± 2.4 µm for DMLS copings and 40.4 ± 6.0 µm for conventionally cast copings. Internal gap values were not reported. The authors concluded that DMLS demonstrated superior marginal adaptation compared with conventional casting [13].

Aydin et al. (2022), in an in vitro study involving 40 Co-Cr restorations, compared SLM, computer-aided design (CAD)-CAM, and conventional lost-wax casting techniques. Vertical misfit values ranged from 105.20 ± 26.22 µm to 167.96 ± 24.1 µm for SLM restorations, 58.80 ± 12.53 µm to 102.71 ± 18.01 µm for CAD-CAM restorations, and 124.81 ± 22.0 µm to 163.30 ± 27.57 µm for lost-wax cast restorations. Horizontal misfit values also varied among fabrication methods. The findings suggested that restoration adaptation was influenced by both fabrication technique and finish-line design [14].

Table 3 summarizes the studies evaluating the effect of DMLS fabrication on the marginal and internal fit of Co-Cr alloy restorations.

**Table 3:** Effect of DMLS on Co-Cr alloys. DMLS: direct metal laser sintering; CAD: computer-aided design; CAM: computer-aided manufacturing; Co-Cr: cobalt-chromium; SLM: selective laser melting; NR: not reported

Author (Year)	Study Design/Sample Size	Alloy Composition/Fabrication Technique	Key Findings	Mean Marginal/Internal Gap (µm)
Bhaskaran et al. (2013) [10]	In vitro comparative study (n=30)	Co-Cr; DMLS vs wax-pattern vs resin-pattern casting	DMLS demonstrated lower marginal discrepancy than conventional techniques	Marginal: wax 45.36; resin 27.22; DMLS 10.52. Internal: wax 39.97; resin 36.15; DMLS 41.22
Arora et al. (2018) [12]	In vitro comparative study (n=30)	Co-Cr; DMLS vs resin-pattern vs wax-pattern casting	DMLS showed lower marginal discrepancy but higher internal discrepancy	Marginal: resin 101.67 ± 4.88; wax 107.84 ± 5.63; DMLS 78.28 ± 6.98. Internal: resin 133.87 ± 6.64; wax 140.84 ± 9.39; DMLS 169.38 ± 6.00
Sharma et al. (2017) [13]	In vitro study (n=30)	Co-Cr; DMLS vs conventional casting	DMLS showed significantly improved marginal adaptation	Marginal: DMLS 27.9 ± 2.4; lost-wax 40.4 ± 6.0. Internal: NR
Aydin et al. (2022) [14]	In vitro study (n=40)	Co-Cr; SLM vs lost-wax casting	Adaptation varied depending on fabrication method and finish-line design	Vertical misfit: SLM 105.20 ± 26.22 to 167.96 ± 24.1; lost-wax 124.81 ± 22.0 to 163.30 ± 27.57. Horizontal misfit: SLM 116.71 ± 27.67 to 137.94 ± 37.85; CAD-CAM 95.31 ± 18.31 to 135.00 ± 13.56; lost-wax 89.38 ± 14.81 to 100.00 ± 13.19

Effect of Conventional Casting on Ni-Cr Alloys

Tjan et al. (1991), in a comparative study involving 40 restorations, evaluated the marginal accuracy of conventionally cast Ni-Cr and titanium crowns. The study demonstrated superior marginal adaptation in Ni-Cr restorations compared with titanium restorations. Exact mean marginal discrepancy values were not available in the accessible source used for this review. Internal gap values were not reported. These findings suggest that Ni-Cr alloys may demonstrate more predictable casting behavior and dimensional stability during conventional fabrication procedures [15].

Table 4 summarizes the studies evaluating the effect of conventional casting techniques on the marginal and internal fit of Ni-Cr alloy restorations.

**Table 4:** Effect of conventional casting on Ni-Cr alloys. Ni-Cr: nickel-chromium; NR: not reported

Author (Year)	Study Design/Sample Size	Alloy Composition/Fabrication Technique	Key Findings	Mean Marginal/Internal Gap (µm)
Tjan et al. (1991) [15]	Comparative study (n=40)	Ni-Cr vs Titanium; lost-wax casting	Ni-Cr demonstrated superior marginal accuracy compared with titanium	Exact mean marginal values are not available in an accessible source. Internal: NR

Effect of DMLS on Ni-Cr Alloys

Konin et al. (2020), in a comparative study involving 30 Ni-Cr restorations, compared DMLS fabrication with induction and centrifugal casting techniques. The study demonstrated significantly improved marginal and internal adaptation in DMLS-fabricated restorations compared with conventionally fabricated restorations. The mean marginal discrepancy was 59.82 ± 5.21 µm for DMLS restorations and 116.13 ± 7.88 µm for induction-cast restorations. Internal discrepancy values were 118.69 ± 20.23 µm for DMLS restorations and 136.94 ± 13.50 µm for induction-cast restorations. Centrifugal casting values were not available in the accessible abstract. These findings suggest that additive manufacturing may reduce dimensional inaccuracies associated with multiple laboratory procedures and improve restoration precision [16].

Table 5 summarizes the study evaluating the effect of DMLS fabrication on the marginal and internal fit of Ni-Cr alloy restorations.

**Table 5:** Effect of DMLS on Ni-Cr alloys. DMLS: direct metal laser sintering; Ni-Cr: nickel-chromium

Author (Year)	Study Design/Sample Size	Alloy Composition/Fabrication Technique	Key Findings	Mean Marginal/Internal Gap (µm)
Konin et al. (2020) [16]	Comparative study (n=30)	Ni-Cr; DMLS vs induction casting vs centrifugal casting	DMLS demonstrated improved marginal and internal adaptation	Marginal: DMLS 59.82 ± 5.21; induction casting 116.13 ± 7.88. Internal: DMLS 118.69 ± 20.23; induction casting 136.94 ± 13.50

Effect of Conventional Casting on Titanium Alloys

Tjan et al. (1991), in a comparative study involving 40 restorations, evaluated conventionally cast titanium and Ni-Cr crowns. The study reported lower marginal accuracy in titanium restorations compared with Ni-Cr restorations. Exact mean marginal discrepancy values were not available in the accessible source used for this review, and internal gap values were not reported. These findings indicate that titanium restorations may demonstrate greater fabrication sensitivity during conventional processing [15].

Table 6 summarizes the studies evaluating the effect of conventional casting techniques on the marginal and internal fit of titanium alloy restorations

**Table 6:** Effect of conventional casting on titanium alloys. Ni-Cr: nickel-chromium; NR: not reported

Author (Year)	Study Design/Sample Size	Alloy Composition/Fabrication Technique	Key Findings	Mean Marginal/Internal Gap (µm)
Tjan et al. (1991) [15]	Comparative study (n=40)	Titanium; lost-wax casting	Titanium demonstrated lower marginal accuracy than Ni-Cr	Exact mean marginal values are not available in an accessible source. Internal: NR

Effect of DMLS on Titanium Alloys

Limited evidence is available regarding DMLS fabrication of titanium restorations because most included titanium studies primarily focused on conventional casting techniques, and only a limited number of investigations evaluated the adaptation of titanium restorations fabricated using the DMLS technique. Therefore, the current evidence is insufficient to draw definitive conclusions regarding the influence of DMLS on the marginal and internal fit of titanium restorations.

Clinical Relevance of DMLS

DMLS has shown improved reproducibility and reduced operator dependency. Studies reported improved marginal adaptation and reduced chairside adjustments with DMLS-fabricated restorations, indicating clinical advantages in workflow efficiency [17-19].

Review-based studies further support these findings. Van Noort (2012) emphasized that digital fabrication techniques consistently demonstrate improved marginal and internal fit compared to conventional methods [19]. Similarly, Revilla-Leon et al. reported that additive manufacturing techniques improve marginal accuracy compared to casting methods [17].

Discussion

This narrative review evaluated the influence of alloy composition and fabrication techniques on the marginal and internal fit of FDPs. The findings presented in Tables 2-6 demonstrate that both alloy characteristics and fabrication workflows influence restoration adaptation and overall prosthesis accuracy.

Traditional fabrication techniques, such as the lost-wax casting technique, are widely used in dentistry to fabricate inlays, onlays, overlays, all-metal crowns, porcelain-fused-to-metal crowns, and components of partial removable dentures. This method enables precise replication of a wax pattern of a prosthetic work by converting a wax model into a metal cast [20]. However, despite the precision offered by the lost-wax casting process, it is susceptible to several potential errors, such as marginal discrepancies and dimensional inaccuracies that may occur due to distortion during wax pattern removal or uneven mold expansion, which can affect the outcome (Figure 1) [21,22]. Meticulous attention to detail is essential during investing and casting to achieve a successful and properly fitting restoration. Incomplete castings, voids, and porosity are other challenges that can arise, particularly if the wax pattern is too thin or if the investment is improperly handled. These issues can compromise the seating of the restoration and may require remaking the prosthesis. Despite its susceptibility to error, the lost-wax casting technique remains advantageous for producing durable, precise, and cost-effective dental restorations when executed correctly [22].

Over time, laser sintering technology has emerged as a popular additive manufacturing system in dentistry. This technology converts CAD data into complex three-dimensional (3D) structures by using a high-powered laser to consolidate powdered material layer by layer. Unlike subtractive milling methods, laser sintering is less time-consuming, minimizes material waste, and reduces labor costs. With the advent of advanced manufacturing techniques, particularly DMLS, the fabrication of dental frameworks has seen significant innovation. DMLS technology, while advanced and widely used in the fabrication of dental prostheses, is susceptible to various errors that can affect the quality and precision of the final product. Common errors in DMLS include the balling phenomenon, where molten metal forms spherical balls instead of smooth layers, leading to rough surfaces and weak inter-layer bonds. Porosity is another issue, often caused by improper laser parameters or inadequate melting, resulting in voids within the material that compromise its strength and fit. Delamination can occur due to residual thermal stresses, causing layers to separate and weakening the structure. Residual stresses are inherent in DMLS due to rapid heating and cooling cycles, potentially leading to warping, cracking, or distortion of the part. Despite these challenges, DMLS offers significant advantages, such as the ability to create highly complex geometries with precision, controlled porosity, and reduced waste material (Figure 2). By carefully controlling process parameters like laser power, scanning speed, and layer thickness, these errors can be minimized, leading to more accurate and durable dental restorations [22,23].

Similarly, in addition to fabrication techniques, alloy composition also plays a significant role in influencing the marginal and internal adaptation of FDPs.

Co-Cr alloys are widely used by clinicians due to their excellent mechanical properties, superior corrosion and wear resistance, and good biocompatibility. The chemical composition of dental Co-Cr alloys consists of 53-67% cobalt (Co), 25-32% chromium (Cr), 2-6% molybdenum (Mo), and small quantities of tungsten (W), silicon (Si), aluminum (Al), and others. Due to the high amount of Cr, a thickness of 1-4 µm passive oxide layer formed on the surface, determining the high corrosion resistance. The good mechanical properties of these alloys are a result of a multiphase structure, age hardening among alloy components, and precipitation of carbides, which substantially increase their hardness. The hardness of the Co-Cr alloys is between 550 and 800 MPa [9,24-26].

The findings summarized in Tables 2, 3 indicate that Co-Cr restorations fabricated using both conventional casting and the DMLS technique generally demonstrated clinically acceptable marginal and internal adaptation. Conventional casting techniques were associated with greater variability in restoration fit because of the multiple technique-sensitive laboratory procedures involved during wax pattern fabrication, investing, burnout, casting, and finishing procedures [15,16]. Although conventionally fabricated restorations demonstrated acceptable adaptation, several studies reported improved marginal adaptation with DMLS and SLM technologies [10-13,27].

The reviewed studies suggest that additive manufacturing techniques may improve restoration precision by reducing manual laboratory procedures that commonly contribute to dimensional inaccuracies during conventional fabrication workflows. Bhaskaran et al. and Sharma et al. both reported superior marginal adaptation with DMLS-fabricated Co-Cr restorations compared with conventional wax-pattern and lost-wax casting techniques [10,13]. These findings support the growing role of the DMLS technique in improving restoration reproducibility and minimizing fabrication-related variability.

However, the reviewed evidence also demonstrated that improved marginal adaptation with DMLS does not necessarily correspond to superior internal adaptation. Arora et al. observed that although DMLS restorations demonstrated lower marginal discrepancies than conventional fabrication methods, internal discrepancy values remained greater in the DMLS group [12]. Similarly, Aydin et al. demonstrated that restoration adaptation varied according to fabrication workflow and finish-line configuration, indicating that scanning accuracy, design parameters, post-processing procedures, and manufacturing settings significantly influence restoration fit [14].

Although Bhaskaran et al. and Arora et al. both reported the lowest marginal discrepancy and the greatest internal discrepancy for DMLS-fabricated copings, the magnitude of the internal gaps differed substantially between the studies. Bhaskaran et al. found only a small difference between DMLS and conventionally fabricated copings, whereas Arora et al. reported a considerably larger and statistically significant internal discrepancy in the DMLS group. This variation may primarily reflect methodological differences. Bhaskaran et al. evaluated all copings sequentially on the same stainless-steel master model, applied a standardized 2-kg seating load with pressure-indicating paste, partially sectioned the copings, and measured internal fit at four predetermined locations [10]. In contrast, Arora et al. seated each coping on its corresponding stone die, sectioned the coping-die assemblies using a computer-controlled water-jet cutter, and evaluated the internal discrepancy on the sectioned specimens. These differences in die material, seating conditions, sectioning procedure, measurement locations, and specimen handling could influence the reported gap values and limit direct numerical comparison. Bhaskaran et al. also attributed the greater internal gap of DMLS copings to manual margin determination and the non-uniform offsetting and shelling algorithms used to generate the coping interior. Arora et al. proposed a similar explanation, noting that finite scanner resolution, rounding of sharp anatomical features, manual margin adjustment, and the offsetting algorithm may increase internal relief despite accurate marginal reproduction. Therefore, the findings suggest that DMLS can produce accurate margins while generating greater internal space, and that the measured magnitude of this discrepancy depends strongly on the digital-design and experimental measurement protocols [12].

Overall, the findings indicate that DMLS technology tends to demonstrate marginal adaptation and fabrication reproducibility in Co-Cr restorations when compared with conventional casting methods. Nevertheless, internal fit and horizontal adaptation may still vary depending on the complete digital workflow, including scanning procedures, software design, finish-line geometry, manufacturing parameters, and post-processing techniques. These observations emphasize that marginal and internal fit should be evaluated independently when determining the clinical acceptability and long-term performance of Co-Cr FDPs.

Ni-Cr alloys are widely used in dentistry for various applications, including the fabrication of crowns, bridges, partial denture frameworks, and orthodontic appliances. The adoption of Ni-Cr alloys in dentistry is attributed to their desirable mechanical properties, good corrosion resistance, low thermal conductivity, biocompatibility, work hardening, machinability, and aesthetics [27]. In general, nickel ranked third among the five most common causes of contact dermatitis, but an investigation regarding toxic reactions to nickel-containing dental alloys shows that the nonprecious dental casting alloys containing up to 84% nickel showed that the concentration of nickel liberated from the metals did not reach cytotoxic levels. Also, very few cases were reported with allergies [28].

The findings summarized in Tables 4, 5 indicate that Ni-Cr restorations generally demonstrated favorable marginal adaptation and dimensional stability when fabricated using both conventional and DMLS techniques [15]. Conventional casting procedures produced clinically acceptable restorations with predictable adaptation, which may be attributed to the favorable casting behavior, mechanical properties, and dimensional stability associated with Ni-Cr alloys [13,18]. Compared with titanium restorations, Ni-Cr restorations consistently demonstrated superior marginal adaptation and reduced fabrication sensitivity during conventional processing procedures [15].

The reviewed studies further demonstrated that additive manufacturing techniques, particularly DMLS, may improve both marginal and internal adaptation of Ni-Cr restorations when compared with conventional casting workflows [16]. DMLS-fabricated restorations generally demonstrated improved restoration precision and reduced dimensional discrepancies, suggesting that this technique may minimize inaccuracies associated with conventional laboratory procedures such as wax pattern fabrication, investing, casting shrinkage, and finishing processes.

Improved adaptation observed in DMLS-fabricated Ni-Cr restorations may be related to the layer-by-layer additive manufacturing process, which reduces manual laboratory variability and improves standardization during framework fabrication. Nevertheless, restoration adaptation remains dependent on multiple workflow-related factors, including scanning accuracy, software design parameters, laser settings, and post-processing procedures. Therefore, although DMLS technology appears to improve restoration reproducibility and adaptation, overall prosthesis fit should still be considered the result of an integrated interaction between alloy properties, manufacturing workflow, laboratory precision, and operator expertise.

Titanium was first identified as a new and unknown metallic element by Gregor in England (1790) and was named titanium several years later (1795) by Klapproth in Germany after the Titans of Greek mythology. Titanium can be found in different combinations for use in dentistry. Pure titanium is composed of 99.5% titanium and 0.5% interstitial elements (carbon, oxygen, nitrogen, hydrogen, and iron), and the proportion of these elements directly affects the metal properties. Commercially pure titanium (CP-Ti) presents similarly to or slightly better than gold alloy types III and IV, Ni-Cr, and Co-Cr normally used in the fabrication of dental frameworks. In the case of pure titanium, besides being an abundant element in nature, it is less toxic than the other materials used in dental prostheses. Moreover, the low density of titanium allows the fabrication of extremely light prostheses. Titanium, in particular, is noted for its excellent biocompatibility and mechanical properties, making it a valuable material in dental prosthetics [29].

The findings summarized in Table 6 indicate that titanium restorations fabricated using conventional techniques demonstrated comparatively greater technique sensitivity and less predictable marginal adaptation than Ni-Cr restorations [15]. Titanium possesses several favorable biological properties, including excellent biocompatibility, corrosion resistance, low density, and reduced allergic potential, making it an important material for dental prostheses. However, despite these advantages, titanium is highly reactive at elevated temperatures and has a high melting point, which may negatively influence casting accuracy and restoration adaptation during conventional laboratory processing procedures [29].

The reviewed studies demonstrated that titanium restorations generally exhibited greater marginal discrepancies and increased fabrication sensitivity when compared with conventionally fabricated Ni-Cr restorations [15]. Tjan et al. reported lower marginal accuracy in titanium restorations compared with conventionally cast Ni-Cr restorations, further supporting the greater technique sensitivity associated with titanium processing [15].

The comparatively reduced adaptation observed in titanium restorations may be related to oxidation reactions, casting shrinkage, contamination during high-temperature processing, and difficulties associated with investing and casting titanium alloys under conventional laboratory conditions. These factors may contribute to increased dimensional inaccuracies and compromise restoration fit. Consequently, although titanium offers significant biological and corrosion-resistant advantages, its processing characteristics may adversely affect marginal adaptation and dimensional stability during conventional fabrication procedures [29].

Overall, the reviewed evidence suggests that restoration adaptation in titanium prostheses is strongly influenced by the interaction between alloy properties, laboratory processing techniques, thermal procedures, and manufacturing precision. Therefore, careful control of casting and ceramic firing procedures is essential to minimize dimensional discrepancies and improve the clinical acceptability of titanium-based restorations.

Limited evidence is available regarding DMLS fabrication of titanium restorations, as most included titanium studies primarily focused on conventional casting techniques. Consequently, direct comparison between DMLS and conventional fabrication methods for titanium restorations remains limited in the current literature.

Overall, the evidence presented in Tables 2-6 suggests that Co-Cr and Ni-Cr alloys appear to provide more predictable marginal adaptation than titanium alloys, while DMLS demonstrates improved reproducibility and marginal accuracy compared with conventional casting methods. Nevertheless, restoration adaptation should be considered the outcome of an integrated workflow involving alloy composition, fabrication technique, laboratory precision, and operator expertise rather than the result of a single material or manufacturing method alone.

Limitations

Most included studies were in vitro, limiting direct clinical extrapolation. Considerable heterogeneity existed among the included studies with respect to study design, measurement techniques, and definitions of acceptable fit, which reduces comparability across studies. In addition, the lack of a universally accepted threshold for marginal and internal fit complicates interpretation. Also, the absence of standardized and universally accepted marginal-fit and internal-fit thresholds made it difficult to interpret the clinical significance of the reported values. A meta-analysis was not feasible because of heterogeneity in alloy types, fabrication techniques, measurement methods, and outcome reporting. Further long-term clinical studies are needed to determine whether improved fit with digital techniques translates into better clinical outcomes.

Overall, the available evidence suggests that Ni-Cr and Co-Cr alloys provide more predictable marginal adaptation than titanium, while the DMLS technique improves reproducibility and reduces variability compared to conventional casting. However, prosthesis fit should be understood as the result of an integrated workflow involving both material selection and fabrication technique. As a narrative review, a formal risk-of-bias assessment and quantitative meta-analysis were not performed.

Clinical implications of the review

The findings of this review indicate that both alloy composition and fabrication technique should be considered together when selecting materials for FDPs, as their interaction influences marginal and internal fit. Current limited evidence suggests that Ni-Cr and Co-Cr alloys generally provide more predictable marginal adaptation, whereas titanium, despite its biocompatibility, may be more technique-sensitive. DMLS offers improved reproducibility and reduced operator dependency, although conventional casting remains clinically acceptable when properly performed. The choice of technique should therefore be guided by clinical requirements, cost, and available resources. Importantly, both marginal and internal fit should be evaluated together to ensure optimal retention, stress distribution, and long-term clinical success.

## Conclusions

This narrative review evaluated the influence of alloy composition and fabrication techniques on the marginal and internal fit of FDPs. Current evidence suggests that both alloy selection and fabrication workflow influence prosthesis adaptation; however, the available evidence is derived predominantly from in vitro studies. Available evidence suggests DMLS may improve reproducibility and marginal accuracy, although clinically acceptable outcomes remain achievable with conventional casting when performed appropriately. Further long-term clinical studies are required to establish whether improved adaptation achieved through digital fabrication methods translates into superior clinical longevity and patient outcomes.
